# Investigation of Differences Between Manufacturers and Public Analyses in Health Technology Assessment in Japan

**DOI:** 10.36469/001c.144530

**Published:** 2025-10-31

**Authors:** Yoko Hirano, Akira Yuasa, Karin Matsumoto, Hiroshi Nakamura

**Affiliations:** 1 Japan Access & Value Pfizer Japan, Inc., Tokyo; 2 Real World Evidence Solutions & HEOR IQVIA Solutions Japan G.K., Tokyo; 3 Graduate School of Business Administration Keio University, Kanigawa, Japan

**Keywords:** health technology assessment, Japan, cost-effectiveness analysis, pricing, incremental cost-effectiveness ratio, quality-adjusted life-year

## Abstract

**Background:**

Japan has a unique drug pricing system that in principle reimburses all regulatory-approved drugs. To ensure sustainability, a health technology assessment (HTA) system was introduced in 2019 to adjust the prices of highly innovative and high-budget-impact drugs based on post-reimbursement cost-effectiveness evaluations.

**Objectives:**

This study aimed to examine the nature and contributing factors of differences between manufacturers’ and public (the Center for Outcomes Research and Economic Evaluation for Health [C2H]) cost-effectiveness analyses for 31 products evaluated under the Japanese HTA system by March 2025.

**Methods:**

We conducted descriptive analyses comparing manufacturers’ and C2H analyses using publicly available reports. Differences in the assessments of additional benefits, incremental cost-effectiveness ratios (ICERs), and reanalysis items were investigated. We explored issues related to orphan drugs and products granted usefulness premiums for attributes not fully captured by quality-adjusted life-years (QALYs), such as improved convenience and prolonged effect.

**Results:**

Among 74 analysis populations across 31 products, 48.6% showed inconsistencies between the manufacturers and C2H in the assessment of additional benefits, outcome measures, or analysis methods used to support those assessments. Inconsistencies in outcome measures and methods increased after the revision of the Japanese HTA system and its guidelines in April 2022. ICER differences were often linked to differences in quality-of-life (QOL) parameters and baseline assumptions. Products granted usefulness premiums for attributes not fully captured by QALYs showed greater ICER differences between the manufacturers and C2H than those without. Although manufacturers often rely on indirect treatment comparisons when evaluating orphan drugs due to limited data and the lack of comparators in clinical trials, these methods were less frequently accepted by C2H due to their associated uncertainty.

**Discussion:**

The findings highlight differences between the manufacturers’ and C2H analyses, including evaluation of QOL, orphan drugs, and attributes not captured by QALYs. Providing clearer guidance, considering other countries’ HTA systems, may help improve consistency in assessments.

**Conclusions:**

This study identified key differences and contributing factors under the Japanese HTA system. The findings are expected to inform future refinements of the system and its guidelines, thereby promoting more transparent and predictable evaluations.

## BACKGROUND

Recent breakthroughs in life sciences have led to the frequent approval and market launch of innovative medicines with no existing treatments.^1–4^ Although these drugs improve clinical outcomes and quality of life (QOL), their high costs place a significant financial burden on healthcare systems. To safeguard public health and ensure efficient use of limited national resources, it is imperative for the healthcare system to evaluate drug value effectively.

In Japan, two regulatory frameworks coexist: (1) the national drug pricing system, which sets reimbursement prices for virtually all approved drugs within 60 days (up to a maximum of 90 days), and (2) the health technology assessment (HTA) system, which adjusts prices based on the results of cost-effectiveness analyses after the drugs have been listed.^5^

Under the Japanese drug pricing system, price premiums may be granted to drugs based on factors such as their innovativeness, pediatric indications, and rapid market introduction, as well as the efficacy and safety they demonstrated in clinical trials during regulatory approval. When these criteria are met, an incentive in the form of a price premium is added to the drug price.^6^

The Japanese HTA system was officially implemented in April 2019 in response to rising healthcare expenditures, following a pilot phase that began in 2016 and initial discussions that started in 2012.^5^ Unlike the HTA systems in other countries such as the United Kingdom (UK), where HTA determines reimbursement eligibility, the Japanese HTA system is used solely to support price adjustments based on cost-effectiveness evaluations after the reimbursement.^7^ It targets products that are considered highly innovative or have a significant financial impact.^5^ Products are categorized into five groups (H1-H5) as follows: H1 includes newly listed products with estimated peak annual sales of at least 10 billion Japanese yen (¥) (approximately US $66.1 million, based on the yearly exchange rate in 2024 reported by the Organization for Economic Co-operation and Development, where US $1 = ¥151.37)^8^; H2 includes newly listed products with estimated peak annual sales of at least ¥5 billion (US $33.0 million) and less than ¥10 billion (US $66.1 million); H3 includes products with exceptionally high prices or those requiring re-evaluation because new indications are added after the analytical frameworks were finalized; H4 includes products listed before the formal implementation of HTA system with annual sales of at least ¥100 billion (US $660.6 million) or those with exceptionally high prices; and H5 includes products whose prices were determined using reference products classified under H1 to H4. The prices of products classified as H5 are automatically adjusted without individual evaluation, using the same price adjustment rates applied to the reference products.^5^

In the Japanese HTA system, the analyses and assessments for individual products are conducted independently by manufacturers and by the public analysis team at the Center for Outcomes Research and Economic Evaluation for Health (C2H).^5^ Once a product is designated for HTA at the time of price listing, the manufacturer and C2H agree on an analytical framework, including analysis populations and comparators. The manufacturer then conducts the analysis and submits the results to both C2H and an expert committee. C2H reviews the submission and, if necessary, performs a reanalysis. Both the manufacturer’s and C2H analyses are subsequently reviewed by the expert committee, which determines the final evaluation.^5^

The assessment begins with a comparison of the additional benefit of the target product relative to a comparator. In the context of the Japanese HTA system, “additional benefit” refers to the added value of the target product relative to the comparators used in the HTA. This concept is distinct from the drug’s efficacy, which is evaluated during the regulatory approval process. Under the HTA framework, if the conclusion is “no additional benefit” or “cannot be determined to have additional benefit,” a cost-minimization analysis is then conducted, resulting in an evaluation of either cost-saving or cost-increasing/equivalent relative to the comparator. Conversely, if an “additional benefit” is determined, a cost-effectiveness analysis is performed, and the final price adjustment rate is determined based on the incremental cost-effectiveness ratio (ICER) value.^5^

Outside Japan, differences between the manufacturers’ analyses and those conducted by public institutions have been reported. For instance, in the UK and Canada, ICERs submitted by manufacturers tend to be lower than those calculated by public bodies, prompting investigations into the causes of these differences.^9,10^

In the Japanese HTA system, the evaluations have been completed for 31 drugs as of March 2025, excluding those classified as H5.^11^ However, no systematic investigation has been conducted into the extent of the differences between the manufacturers’ and C2H analyses, nor into the contributing factors.

Additionally, we have identified two key issues for discussion within the current Japanese HTA system. First, it remains unclear whether products granted usefulness premiums for attributes such as improved convenience, positioning as a standard treatment, prolonged effect, and reduced invasiveness—factors that are not fully captured by the quality-adjusted life-year (QALY)—should be evaluated using the same criteria as other products. According to the Japanese HTA guidelines, QOL values used to calculate QALYs should primarily be measured using the Japanese version of the EuroQol 5-Dimension 5-Level (EQ-5D-5L),^12^ which assesses mobility, self-care, usual activities, and pain/discomfort.^13^ These domains may not fully reflect improvements in convenience or similar attributes.

Second, it is uncertain whether it is appropriate to evaluate orphan drugs using the same criteria as other products given that these drugs are often approved based on limited data and may involve substantial analytical uncertainty.

Therefore, this study aimed to examine the nature and contributing factors of differences between the manufacturers’ and C2H analyses, with the goal of informing improvements to the Japanese HTA system and its guidelines. Furthermore, we investigated evaluation practices related to the two issues described above.

## METHODS

### Overview

This study included 31 drugs classified as H1 through H4 whose evaluation results had been publicly disclosed between the introduction of the system in April 2019 and March 2025. Products classified as H5, as well as medical devices, were excluded from the analysis. The results of the manufacturers’ analyses and those of C2H were compared and descriptively summarized.

### Data Collection

Data were collected from the manufacturers’ and C2H analytical reports listed on the C2H website,^11^ and price adjustment notifications published by Central Social Insurance Medical Council (Chuikyo).^14,15^ For products whose manufacturers’ analytical reports were not disclosed due to the manufacturers’ preferences, the corresponding information was obtained from the C2H analytical reports. Four reviewers (Y.H., K.M., H.M., and Y.L.) independently extracted the data. To ensure accuracy, each dataset was independently cross-checked by a reviewer other than the original extractor. Final verification of data consistency was conducted by Y.H., A.Y., and H.N.

### Evaluation Criteria and Analytical Method

**Comparison between the manufacturers’ and C2H assessments of additional benefits:** In the Japanese HTA system, the first step involves assessing the additional benefit of the target product compared to the comparator.^12^ In this study, we separately collected data on the assessments of additional benefits and on the outcome measures and analysis methods used to support these assessments, for all analysis populations across 31 products, as conducted by the manufacturers and by C2H, respectively.

Based on the collected information, the analysis populations were classified into 4 categories depending on whether the assessments of additional benefits, as well as the outcome measures and analysis methods used, were consistent or inconsistent between the manufacturers and C2H.

In April 2022, revisions were made to the Japanese HTA system and its associated guidelines.^16^ Accordingly, in addition to analyses covering the entire period, we conducted stratified analyses by evaluation period: products designated for HTA from April 2019 to March 2022 (early period), and those designated from April 2022 to March 2025 (late period).

For the analysis populations where the assessments of additional benefits differed between the manufacturers and C2H, the specific factors contributing to the differences were investigated. When multiple factors were identified, the one deemed most influential—based on descriptions in the C2H analytical reports—was selected. If the same factor was identified across multiple populations within a single product, it was counted only once.

**Comparison of ICERs between the manufacturers’ and C2H analyses, and items subject to reanalysis:** In the Japanese HTA system, the final price adjustment rate is determined based on the ICER value.^5^ When multiple analysis populations are defined for a single product, the final price adjustment rate is calculated as a weighted average based on their respective patient proportions. In cases where no additional benefit is identified, or where an additional benefit cannot be determined, a cost-minimization analysis is applied and calculation of the ICER is not feasible. Therefore, in this study, the cost-effectiveness results were categorized into 4 categories based on the ICER thresholds used in the Japanese HTA system for the price adjustment.^5^ In addition, the cutoff values in parentheses for each category were applied to products requiring special consideration in a comprehensive assessment—such as drugs for intractable diseases with limited treatment options, pediatric conditions, or cancer drugs. The 4 categories defined in this study are as follows:

- **Category 1**: Less than ¥5 million [US $33 032]/QALY (or less than ¥7.5 million [US $49 547]/QALY), dominant, or cost-saving
- **Category 2:** At least ¥5 million [US $33 032]/QALY and less than ¥7.5 million [US $49 547]/QALY (or at least ¥7.5 million [US $49 547]/QALY and less than ¥11.25 million [US $74 321]/QALY)
- **Category 3:** At least ¥7.5 million [US $49 547]/QALY and less than ¥10 million [US $66 063]/QALY (or at least ¥11.25 million [US $74 321]/QALY and less than ¥15 million [US $99 095]/QALY)
- **Category 4:** At least ¥10 million [US $66 063]/QALY (or at least ¥15 million [US $99 095]/QALY), or cost-increasing/equivalent.

In this study, a difference of 2 or more levels in the ICER category between the manufacturer and C2H was defined as a “major difference.” Differences such as “dominant” vs “other results,” or “cost-saving or cost-increasing/equivalent” vs “other results” between the manufacturer and C2H, were also considered a “major difference” even when the ICER category varied within 1 level. For each product, we examined the items within the manufacturer’s analyses that C2H deemed to require reanalysis. We compared the average number of items between products with and without major differences and examined the nature of these items. Products with any analysis population evaluated using cost-minimization analysis were excluded, as C2H often grouped issues under “implementation of cost-minimization analysis.”

The items requiring reanalysis were classified into 8 categories: (1) model settings, (2) baseline parameter settings such as patient background and epidemiological data, (3) parameter settings related to the proportion of patients in each health state or transition probabilities in the model, (4) parameter settings related to outcomes associated with drug administration, (5) parameter settings related to QOL, (6) parameter settings related to costs, (7) other parameter settings, and (8) incorporation of the latest drug prices and medical service fees. Given that revisions to drug prices and medical service fees are conducted systematically and at regular intervals, the results may be influenced by the timing of their evaluation. Therefore, we also conducted an analysis excluding category (8).

It is noted that in the Japanese HTA system, the price adjustment rate is determined not only by the results of the cost-effectiveness evaluation but also by the initial premium rate granted at the time of drug approval.^5^ As a result, simple comparisons of price adjustment rates across products are not appropriate. Therefore, this study does not include product-specific price adjustment rates.

**Comparison of ICERs between products with and without usefulness premiums granted for attributes not fully captured by QALYs**: Among the reasons for awarding usefulness premium in the Japanese drug pricing system, the following are considered not fully captured by QALY: (1) improved convenience, (2) positioning as a standard treatment, (3) prolonged effect, and (4) reduced invasiveness. This study compared ICERs between the manufacturers’ and C2H analyses for products with and without such premiums. When a single ICER could not be determined for a single product due to multiple analysis populations or cost-minimization results, each population was categorized into 1 of the 4 ICER categories previously described in the **Methods**. A weighted ICER score per product was calculated by multiplying the ICER category (Categories 1-4) of each analysis population by its corresponding patient proportion and summing the results. The patient proportions were obtained from the price adjustment notifications published by Chuikyo^14,15^ in Japan. For example, if one analysis population is in ICER Category 1 and accounts for 60% of patients, and another is in ICER Category 2 and accounts for 40%, the ICER score for this product is calculated as 1 × 0.6 + 2 × 0.4 = 1.4. Summary statistics were presented using box-and-whisker plots.

**Comparison of uncertainty between orphan and non-orphan drugs**: This study examined whether there were differences in analytical uncertainty between orphan drugs and non-orphan drugs, within the scope of Japanese HTA. As an indicator of uncertainty, we investigated whether an indirect treatment comparison (ITC) was used for the assessments of additional benefits by both the manufacturers and C2H.

Although drugs used exclusively for designated intractable diseases are exempt from the Japanese HTA system, products with high estimated peak sales (>¥35 billion [US $231.2 million]) or high unit prices may still be designated for HTA, depending on decisions by Chuikyo.^14,15^ Therefore, we conducted a separate analysis excluding drugs for designated intractable diseases from the orphan drug group. In the C2H analysis, if C2H either accepted the manufacturer’s ITC methods and results or conducted its own ITC, the analysis was considered to involve an ITC. Additionally, a separate aggregation was conducted, excluding products for which comparative clinical trials against the HTA-designated comparators had been conducted for regulatory approval.

### Data Analysis

Data aggregation and analysis were performed using Microsoft Excel and R version 2024.12.1. The box-and-whisker plots were created using ggplot2.

## RESULTS

### Background and Characteristics of the Target Products

The background and characteristics of the target products are shown in **Table 1**. A total of 31 products included 74 analysis populations. Of these, 17 products (54.8%) and 14 products (45.2%) were designated for HTA from April 2019 to March 2022 (early period) and from April 2022 to March 2025 (late period), respectively. The indications varied widely, including but not limited to cancer, respiratory diseases, and COVID-19. Most of the products (83.9%) were classified under HTA category H1, which corresponds to estimated peak annual sales of at least ¥10 billion (US $66.1 million). In the manufacturers’ analyses, 71.6% of the analysis populations were evaluated as having favorable cost-effectiveness (<¥5 million [US $33 032]/QALY for normal products or <¥7.5 million [US $49 547]/QALY for products requiring special consideration, dominant, or cost-saving), whereas in the C2H analyses, this proportion was 43.2%.

**Table 1.** Background and Characteristics of Target Products

	**No. (%) of Products (N = 31)**	**No. (%) of Analysis Populations (N = 74)^a^**
Timing of HTA designation		
April 2019–March 2022 (early period)	17 (54.8)	51 (68.9)
April 2022–March 2025 (late period)	14 (45.2)	23 (31.1)
Indications^b^		
Cancer^c^	11 (33.3)	19 (25.7)
Respiratory diseases	4 (12.1)	17 (23.0)
Digestive system diseases	1 (3.0)	4 (5.4)
Renal and urological diseases	2 (6.1)	2 (2.7)
Circulatory system diseases	2 (6.1)	2 (2.7)
COVID-19	3 (9.1)	6 (8.1)
Diabetes	2 (6.1)	4 (5.4)
Other diseases	11 (33.3)	20 (27.0)
Classification of HTA		
H1: Estimated peak annual sales: ≥¥10 billion (US $66.1 million)^d^	26 (83.9)	–
H2: Estimated peak annual sales: ≥¥5 billion (US 33.0million)and<¥10billion(US66.1 million)^d^	4 (12.9)	–
H3: Exceptionally high prices or re-evaluation is required because new indications are added	1 (3.2)	–
H4: Listed before the formal implementation of HTA system with annual sales of ≥¥100 billion (US $660.6 million)^d^ or with exceptionally high prices	0 (0)	–
ICER categories^e^ in manufacturers’ analyses		
Category 1: <¥5 million/QALY (or <¥7.5 million/QALY), dominant, or cost-saving	–	53 (71.6)
Category 2: ≥¥5 million/QALY and <¥7.5 million/QALY (or ≥¥7.5 million/QALY and <¥11.25 million/QALY)	–	6 (8.1)
Category 3: ≥¥7.5 million/QALY and <¥10 million/QALY (or ≥¥11.25 million/QALY and <¥15 million/QALY)	–	2 (2.7)
Category 4: ≥¥10 million/QALY (or ≥¥15 million/QALY), or cost-increasing/equivalent	–	11 (14.9)
Not analyzable	–	2 (2.7)
ICER categories^e^ in C2H analyses		
Category 1: <¥5 million/QALY (or <¥7.5 million/QALY), dominant, or cost-saving	–	32 (43.2)
Category 2: ≥¥5 million/QALY and <¥7.5 million/QALY (or ≥¥7.5 million/QALY and <¥11.25 million/QALY)	–	9 (12.2)
Category 3: ≥¥7.5 million/QALY and <¥10 million/QALY (or ≥¥11.25 million/QALY and <¥15 million/QALY)	–	3 (4.1)
Category 4: ≥¥10 million/QALY (or ≥¥15 million/QALY), or cost-increasing/equivalent	–	26 (35.1)
Not analyzable	–	4 (5.4)
Products with usefulness premiums^f^		
Novel mechanism of action with clinical utility	9 (29.0)	–
Higher efficacy or safety compared to similar drugs	8 (25.8)	–
Improvement in treatment for the target disease	18 (58.1)	–
Significant clinical utility derived from formulation improvements	1 (3.2)	–
Products without usefulness premiums^g^	6 (19.4)	–
Orphan drugs	8 (25.8)	–
Orphan drugs for designated intractable diseases	3 (9.7)	–
Orphan drugs not for designated intractable diseases	5 (16.1)	–
Non-orphan drugs	23 (74.2)	–

Abbreviations: C2H, Center for Outcomes Research and Economic Evaluation for Health; HTA, health technology assessment; ICER, incremental cost-effectiveness ratio; QALY, quality-adjusted life-year.

^a^One analysis population was excluded from the HTA due to lower effectiveness compared to the comparator and was therefore not included in this study.

^b^When a single product had multiple indications, each indication was counted separately. As a result, the total number of indications exceeds the number of target products. Indications in the same category (eg, breast cancer and gastric cancer) for a single product were counted separately.

^c^Malignant neoplasms were classified under the “cancer” category.

^d^The yearly exchange rate in 2024 reported by Organisation for Economic Co-operation and Development is US $1 = ¥153.73.

^e^The cutoff values in parentheses for each category were applied to products requiring special consideration in a comprehensive assessment, such as drugs for intractable diseases with limited treatment options, pediatric conditions, or cancer drugs. The following are the 4 categories denominated in US dollars based on the yearly exchange rate in 2024: Category 1: <US $33 032/QALY (or <US $49 547/QALY), dominant, or cost-saving; Category 2: ≥ US $33 032/QALY and < US $49 547/QALY (or ≥US $49 547/QALY and <US $74 321/QALY); Category 3: ≥ US $49 547/QALY and <US $66 063/QALY (or ≥US $74 321/QALY and <US $99 095/QALY); and Category 4: ≥US $66 063/QALY (or ≥US $99 095/QALY), or cost-increasing/equivalent.

^f^When a single product included multiple usefulness attributes, each was counted separately. As a result, the total number of products with usefulness premiums exceeds the number of target products.

^g^Products with disclosure rates <50% in the cost calculation method were included.

**Figure 1.**
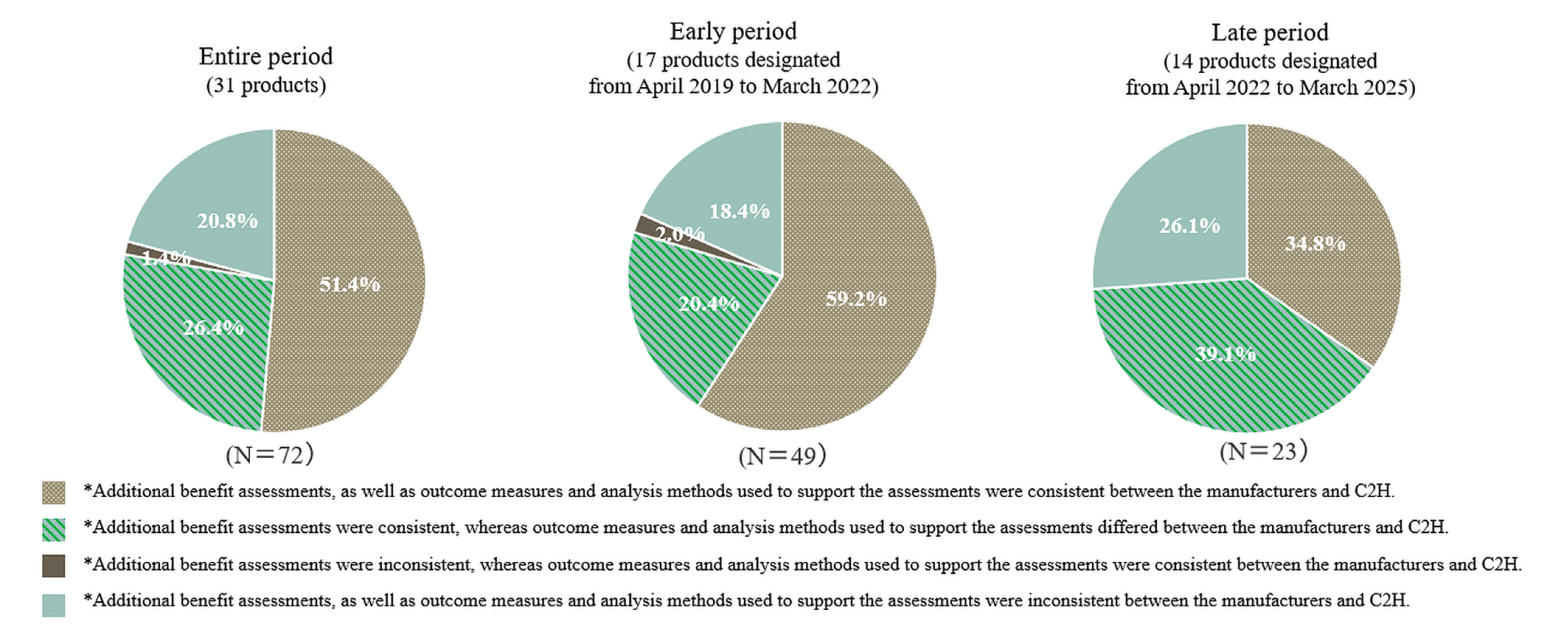
Proportions of Consistency and Inconsistency in Additional Benefits, Outcome Measures, and Analysis Methods Over Time Abbreviation: C2H, Center for Outcomes Research and Economic Evaluation for Health. *Two analysis populations deemed unanalyzable by both the manufacturers and C2H were excluded.

**Comparison between the manufacturers’ and C2H assessments of additional benefits**: The proportion of analysis populations showing inconsistencies between the manufacturers and C2H, whether in the assessment of additional benefits, outcome measures or analysis methods used to support those assessments, was 48.6% for the entire period, 40.8% for the early period, and 65.2% for the late period (**Figure 1**). In contrast, the proportions demonstrating full consistency across these elements were 51.4% (entire period), 59.2% (early period), and 34.8% (late period), indicating a decline in consistency in the later period.

**Supplementary Table S2** shows the breakdown of consistency and inconsistency in the assessments of additional benefits as well as the outcome measures and analysis methods used to support the assessments. Within the analysis populations with consistent assessments of additional benefits between the manufacturers and C2H, the proportion of the population that showed inconsistency in the outcome measures or analysis methods used to support the assessments was 33.9% for the entire period, 25.6% for the early period, and 52.9% for the late period. On the other hand, among the analysis populations with inconsistent assessments of additional benefits, nearly all populations had different outcome measures or analysis methods used to support the assessments (93.7% for the entire period, 90.0% for the early period, and 100% for the late period).

To further investigate these variations, we conducted an analysis based on product indications. Across the entire period, the proportion of analysis populations demonstrating full consistency in the assessment of additional benefits, outcome measures and analysis methods was 73.7% for oncology products (7 products, 19 analysis populations) compared with 43.4% for non-oncology products (24 products, 53 analysis populations). All oncology products in the present study were included in the early period, during which the full consistency rate was at 73.7%, while the rate for non-oncology products (10 products, 30 analysis populations) was 50.0%. Among the analysis populations with consistent assessments of additional benefits between the manufacturers and C2H, the proportion that showed inconsistencies in the outcome measures or analysis methods used to support the assessments was 22.2% for oncology products (early period only) and 28.6% and 52.9% for non-oncology products in the early and late periods, respectively.

For the 16 analysis populations with inconsistent assessments of additional benefits between the manufacturers and C2H, the contributing factors to these inconsistencies are presented in **Table 2**. The most frequent factor in the entire period was “differences in the trials used for evaluation” (27.3%), followed by “differences in the outcome measures used for evaluation,” “differences in the analytical methods,” “differences in the interpretation of results,” and “deviations from analytical guidelines or frameworks” (each accounting for 18.2%).

**Table 2.** Factors Contributing to Inconsistencies in Assessments of Additional Benefits Between Manufacturers and C2H

**Factors Contributing to Inconsistencies**	**Entire Period, n (%) (N=11)^a^**	**Early Period, n (%) (N=6)**	**Late Period, n (%) (N=5)**
Differences in trials used for evaluation	3 (27.3)	2 (33.3)	1 (20.0)
Differences in outcome measures used for evaluation	2 (18.2)	1 (16.7)	1 (20.0)
Differences in analytical methods	2 (18.2)	2 (33.3)	0 (0)
Differences in interpretation of results	2 (18.2)	1 (16.7)	1 (20.0)
Deviations from analytical guidelines or frameworks	2 (18.2)	0 (0)	2 (40.0)

Abbreviation: C2H, Center for Outcomes Research and Economic Evaluation for Health.

^a^If the same factor was identified across multiple populations within a single product, it was counted only once. Among the 16 analysis populations with inconsistent assessments of additional benefits between the manufacturers and C2H, 11 cases were identified.

**Comparison of ICERs between the manufacturers’ and C2H analyses, and items subject to reanalysis**: This analysis included 17 products, excluding 13 products for which cost-minimization analysis was conducted in at least 1 analysis population, and 1 product for which 1 analysis population was ultimately excluded from the HTA.

Among the analyzed products, 6 products showed a major difference in ICERs between the manufacturer and C2H—defined as a difference of 2 or more ICER categories, or a combination of “dominant” vs “other results,” or “cost-saving or cost-increasing/equivalent” vs “other results” in at least 1 analysis population within a single product—while the remaining 11 products did not (**Table 3**). The average number of items identified as requiring reanalysis was 3.50 for the group with major differences and 1.73 for the other group, indicating that products with greater ICER differences tended to have more items flagged for reanalysis. This trend remained consistent even when the item “incorporations of the latest drug prices and medical service fees” was excluded, with averages of 2.67 and 1.55, respectively.

**Table 3.** Number and Nature of Reanalysis Items by ICER Difference Between Manufacturers’ and C2H Analyses

**Category**	**Products With Major Differences in ICERs^a^ (N = 6)^b^**	**Products Without Major Differences in ICERs^a^ (N = 11)**	**Total (N = 17)^c^**
Average No. of items requiring reanalysis (*including* incorporation of the latest drug prices and medical service fees)	3.50	1.73	2.35
Average No. of items requiring reanalysis (*excluding* incorporation of the latest drug prices and medical service fees)	2.67	1.55	1.94
**Products requiring reanalysis, n (%)**			
Model settings	0 (0)	1 (9.1)	1 (5.9)
Baseline parameter settings (eg, patient background, epidemiological data)	3 (50.0)	2 (18.2)	5 (29.4)
Parameter settings related to the proportion of patients in each health state or transition probabilities in the model	2 (33.3)	1 (9.1)	3 (17.6)
Parameter settings related to outcomes associated with drug administration	4 (66.7)	7 (63.6)	11 (64.7)
Parameter settings related to QOL	4 (66.7)	4 (36.4)	8 (47.1)
Parameter settings related to costs	2 (33.3)	1 (9.1)	3 (17.6)
Other parameter settings	1 (16.7)	1 (9.1)	2 (11.8)
Incorporation of the latest drug prices and medical service fees	5 (83.3)	2 (18.2)	7 (41.2)

Abbreviations: C2H, Center for Outcomes Research and Economic Evaluation for Health; ICER, incremental cost-effectiveness ratio; QOL, quality of life.

^a^In this study, a difference of ≥2 levels in the ICER category between the manufacturer and C2H was defined as a “major difference.” Differences such as “dominant” vs “other results”, or “cost-saving or cost-increasing/equivalent” vs “other results” between the manufacturer and C2H, were also considered a “major difference” even when the ICER category varied within 1 level.

^b^One product, for which both a cost-effectiveness analysis and a cost-minimization analysis were conducted for one analysis population in the C2H evaluation, was included.

^c^One product was excluded from this analysis because one of the analysis populations had been excluded from the HTA due to lower effectiveness compared with the comparator.

Among the items requiring reanalysis, the most frequently cited were parameter settings related to outcomes associated with drug administration (64.7%); parameter settings related to QOL (47.1%); and incorporation of the latest drug prices and medical service fees (41.2%) (**Table 3**). Three items showed at least 30% higher frequency in the group with major differences in ICERs compared with that without: incorporation of the latest drug prices and medical service fees (83.3% vs 18.2%); baseline parameter settings (50.0% vs 18.2%); and parameter settings related to QOL (66.7% vs 36.4%).

Additionally, the analysis that grouped the product with a one-level difference in the ICER category between the manufacturer and C2H (N=1) together with those showing broader ICER differences demonstrated results consistent with those presented in **Table 3**.

**Figure 2.**
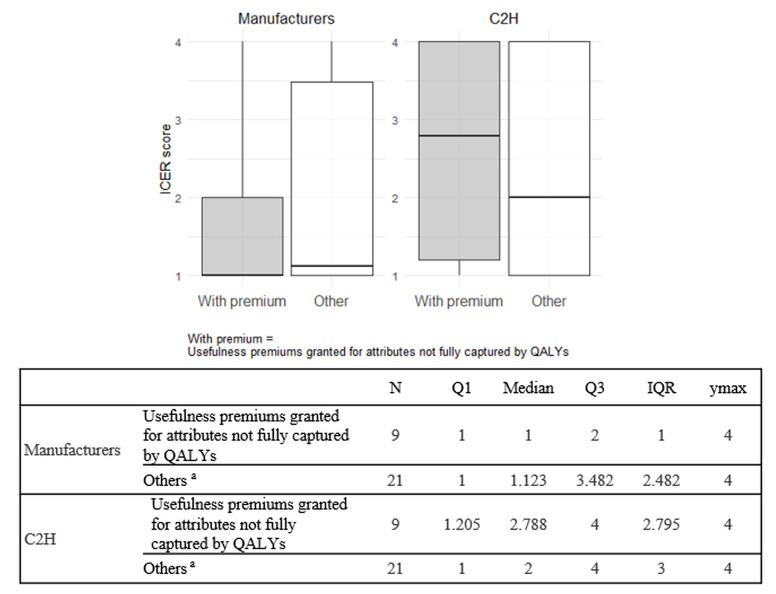
ICER Score Differences by Usefulness Premium Status for Attributes Not Captured by QALYs Abbreviations: C2H, Center for Outcomes Research and Economic Evaluation for Health; ICER, incremental cost-effectiveness ratio; IQR, interquartile range; QALY, quality-adjusted life-year. The ICER score for each product was calculated by multiplying the ICER category (Category 1 to 4) of each analysis population by its corresponding patient proportion and summing the results. ^a^One product was excluded because an ICER score could not be calculated due to the lack of disclosed patient proportions by analysis population.

**Comparison of ICERs between products with and without usefulness premiums granted for attributes not fully captured by QALYs:** Nine products received usefulness premiums for attributes not fully captured by QALYs. Summary statistics of ICER scores for both the manufacturers and C2H were calculated for products with and without such usefulness premiums and are presented in **Figure 2**.

The ICER scores in C2H analyses (medians, 2.788 and 2) were higher than those in the manufacturers’ analyses (medians, 1 and 1.123) for both products with and without usefulness premiums granted for attributes not fully captured by QALYs. The differences in ICER scores between the manufacturers’ and C2H analyses were greater for products with such premiums (median difference, 1.788) than for those without (median difference, 0.877). Furthermore, in the manufacturers’ analyses, there was no notable difference in median ICER scores between the two groups (1 vs 1.123). On the other hand, in C2H analyses, products with usefulness premiums granted for attributes not fully captured by QALYs had higher median ICER scores compared to those without (2.788 vs 2).

**Comparison of uncertainty between orphan and non-orphan drugs**: A total of 8 products were classified as orphan drugs, 3 of which also met the criteria for designated intractable diseases. The inclusion of ITCs was assessed for both orphan and non-orphan drugs in the manufacturers’ and C2H analyses. The results are presented in **Table 4**.

**Table 4.** Inclusion of ITCs for Orphan and Non-orphan drugs in Manufacturers’ and C2H Analyses

**Inclusion of ITC**	**Manufacturers**	**C2H^a^**	**C2H vs Manufacturers^b^**
Orphan drugs (N = 8)	50.0% (4/8 products)	25.0% (2/8 products)	50.0% (2/4 products)
Orphan drugs excluding those for designated intractable diseases (N = 5)	60.0% (3/5 products)	40.0% (2/5 products)	66.7% (2/3 products)
Non-orphan drugs (N = 23)	39.1% (9/23 products)	39.1% (9/23 products)	88.9% (8/9 products)^c^

Abbreviations: C2H, Center for Outcomes Research and Economic Evaluation for Health; ITC, indirect treatment comparison.

^a^Products were included where C2H either accepted the methods and results of ITCs conducted by the manufacturers or conducted ITCs independently.

^b^Proportion of products for which C2H either accepted the manufacturers’ ITCs or conducted ITCs independently, among those where the manufacturers included such analyses.

^c^The denominator represents the number of products for which ITCs were conducted in the manufacturers’ analyses. One product was not included in the numerator, for which C2H conducted an ITC while the manufacturer did not.

Among orphan drugs, the proportion of products for which ITCs were conducted to assess the additional benefits was 50.0% in the manufacturers’ analyses. On the other hand, in the C2H analyses, the proportion of products where either the manufacturers’ ITC methods and results were accepted, or ITCs were conducted independently was 25.0%. In contrast, among non-orphan drugs, the corresponding proportions were 39.1% in both the manufacturers’ and C2H analyses.

Among products for which ITCs were conducted in the manufacturers’ analyses, the proportion for which C2H either accepted the methods and results or conducted them independently was lower for orphan drugs (50.0%) compared with non-orphan drugs (88.9%). This trend remained consistent even when excluding orphan drugs for designated intractable diseases.

Furthermore, a similar trend was observed even when excluding products for which comparative clinical trials against the HTA-designated comparators had been conducted for regulatory approval (see **Supplementary Table S3**). Among orphan drugs, 3 products were approved based on single-arm trials without comparators, and 1 product used a placebo as the comparator. For all 4 of these products, ITCs were conducted in the manufacturers’ analyses. However, C2H accepted the methods and results of the manufacturers’ ITCs or conducted them independently in only 2 of the 4 products (50.0%). In contrast, the corresponding proportion among non-orphan drugs was 87.5%.

## DISCUSSION

This study investigated the nature and contributing factors of differences between the manufacturers’ and C2H analyses for 31 HTA-designated products whose evaluation results had been publicly disclosed as of March 2025.

The proportion of analysis populations showing inconsistencies between the manufacturers and C2H in the assessment of additional benefits, outcome measures or analysis methods used to support those assessments, increased after the revision of the HTA guidelines in April 2022. Oncology products, all of which were included in the early period, showed a high level of consistency between the manufacturers and C2H, contributing to the overall consistency rate observed during that period. Even when oncology products were excluded, inconsistency rates increased in the later period, particularly in outcome measures or analysis methods used to support the assessments of additional benefits. One possible explanation for this trend is the accumulation of case-based experience by manufacturers and their increasing efforts to assess additional benefits using novel indicators and methodological approaches. While analyses are, in principle, expected to adhere to the HTA guidelines, alternative approaches may be necessary in some cases due to disease characteristics or drug-specific factors that make strict adherence challenging. For example, although the HTA guidelines recommend using randomized clinical trial data to assess additional benefits, in diseases such as COVID-19, where emerging variants alter disease characteristics, manufacturers have argued that the most recent real-world evidence would be more appropriate.^17,18^ Furthermore, although the guidelines designate the EQ-5D-5L as the preferred measure for QOL, in cases where it is considered inadequate for capturing the disease state, manufacturers have advocated for the use of alternative measures better suited to specific disease characteristics, prompting further discussion.^19,20^ As such, when alternative measures are used, it is important that manufacturers provide clear rationales and that public institutions consider such cases with appropriate flexibility.

Additionally, this study showed that inconsistencies in parameter settings related to QOL were assumed to contribute to differences in ICERs. A detailed review of QOL measures used in the 31 products^11^ revealed that, excluding those evaluated using cost-minimization analysis, the proportion of products deemed to require reanalysis regarding QOL was 25.7% (2/7 products) for those utilizing EQ-5D as the QOL measure, compared with 70.0% (7/10 products) for those using non-EQ-5D measures. These findings suggest that using EQ-5D, the first-choice QOL measure recommended in the HTA guidelines,^12^ is associated with a lower likelihood of requiring reanalysis. In contrast, 10 of the 31 products employed the vignette method, a lower-priority QOL measure per the guidelines,^12^ and it was accepted by C2H in only 2 products (20.0%). Given that the optimal QOL measure for cost-effectiveness analysis may vary depending on disease characteristics and available clinical trial data, a more flexible evaluation approach is warranted.

A comparison of ICER scores between the manufacturers and C2H for products granted usefulness premiums for attributes not fully captured by QALYs revealed greater differences in median ICERs than for those without such premiums. A detailed review showed that none of the products receiving premiums for attributes such as improved convenience, positioning as a standard treatment, prolonged effect, and reduced invasiveness were judged by C2H to have additional benefits based on QOL improvements. For example, in one product, a manufacturer claimed that extended dosing intervals improved QOL, justifying a premium for prolonged effect; however, this claim was not accepted by C2H. Furthermore, this product was evaluated as having “no additional benefit” or “cannot be determined to have additional benefit” because the supporting clinical trial was a non-inferiority study.^21^ In contrast, the National Institute for Health and Care Excellence (NICE) explicitly considers patient convenience, including process improvements not captured in cost analysis.^22^ For the same product, NICE conducted a cost-effectiveness analysis, found favorable results, and issued a recommendation based on potential QOL gains from extended dosing intervals.^23^ Under the current Japanese HTA system, price adjustments are applied to the premium portion granted for additional usefulness. However, when the rationale for such premiums cannot be adequately reflected in the assessment of additional benefits or cost-effectiveness analysis, applying the same evaluation criteria as for other products may raise fairness concerns and warrants further discussion.

Among the products for which manufacturers conducted ITCs, the proportion of products in which the methods and results of those ITCs were accepted or in which independent ITCs were conducted by C2H was lower for orphan drugs than for non-orphan drugs. This suggests that, while ITCs are often necessary for orphan drugs due to limited data and the absence of comparators in clinical trials, the inherent uncertainty associated with such limited data reduces the likelihood of their acceptance by C2H. These findings highlight limitations in evaluating the cost-effectiveness of orphan drugs under the current HTA system in Japan.

According to a review investigating how drugs for rare diseases are evaluated within the HTA systems of Oceania (Australia and New Zealand) and Europe including the UK (England, Scotland, and Wales), France, Germany, Italy, Spain, Sweden, and the Netherlands, all of these countries consider factors such as disease severity, lack of alternative treatments, therapeutic value, quality of evidence, and cost-effectiveness.^24^ Many of these countries apply flexible evaluation criteria for rare diseases. Among these, all countries except the UK (specifically NICE) do not apply clearly defined ICER thresholds for the evaluation of the drugs for rare diseases.^24^ In contrast, NICE’s Highly Specialised Technologies program allows a relaxed cost-effectiveness threshold^24^ for drugs that meet specific criteria,^25^ such as being indicated for ultra-rare conditions (prevalence of ≤1:50 000), lifelong diseases with significant burden on patients and families, and offering substantial additional benefits over existing treatments. In Australia, even if drugs are not deemed cost-effective, it may be reconsidered under the Life Saving Drugs Program if they meet certain criteria, such as being indicated for ultra-rare conditions (prevalence of ≤1:50 000), imposing significant burden on patients and families, demonstrating clinical effectiveness, and lacking alternative treatment options.^24,26^ In the Japanese HTA system, drugs indicated exclusively for designated intractable diseases are exempt from assessment, and a more relaxed ICER threshold is applied to drugs that include such indications among others.^5^ However, no special consideration is currently afforded to orphan drugs. The present study demonstrated that the evaluation of orphan drugs is generally associated with greater uncertainty and is more challenging compared to that of non-orphan drugs. Therefore, the appropriateness of evaluating orphan drugs under the current Japanese HTA system requires careful consideration. If such evaluations are to be conducted, it may be necessary to adopt more flexible approaches such as accepting alternative evaluation methods or applying a more relaxed ICER threshold considering the inherent uncertainties.

In addition, under the Japanese HTA system, a cost-minimization analysis is conducted when a product is assessed as having “no additional benefit” or “cannot be determined to have additional benefit” compared with the comparators. The appropriateness of conducting a cost-minimization analysis has been raised as a concern by Briggs et al,^27^ and further discussion is needed regarding the continued use of such approach within the current Japanese HTA framework.

There are some limitations in this study. First, for products without disclosed manufacturer analysis reports, information was sourced from the C2H analysis reports. Although we could not fully examine the manufacturers’ analyses, the C2H reports included the rationales for the assessments of additional benefits, evaluation methods, cost-effectiveness results, and items requiring reanalysis by C2H. Therefore, the impact of this limitation is considered minimal.

Second, the number of cases included in this study was limited, as it focused on 31 products whose evaluation results had been publicly disclosed by March 2025. In addition, subgroup analyses by evaluation period and the exclusion of exceptional cases further reduced the sample sizes. Given that further revisions to the HTA system and its guidelines are anticipated in Japan, continued accumulation of cases and validation of the findings presented in this study will be necessary.

Third, the calculation of product-specific ICER scores using the ICER thresholds defined in the Japanese HTA system was uniquely developed for the purposes of this study.

## CONCLUSIONS

This study elucidated the differences between the manufacturers’ and C2H analyses under the Japanese HTA system, along with the underlying contributing factors. The findings are expected to inform future refinements of the system and its guidelines, thereby promoting more transparent and predictable evaluations.

### Disclosures

Y.H. and A.Y. are full-time employees of Pfizer Japan Inc. K.M. is a full-time employee of IQVIA Solutions Japan G.K., which received funding from Pfizer Japan Inc. to conduct this study. H.N. has received consulting fees from Pfizer Japan Inc.

## Supplementary Material

Online Supplementary Material

## Data Availability

The datasets generated during and/or analyzed during the current study are available from the corresponding author on reasonable request.
