# Supplementary material for: Investigation of Differences Between Manufacturers and Public Analyses in Health Technology Assessment in Japan
Source: J Health Econ Outcomes Res. 2025 Oct 31;12(2):173–82. doi: 10.36469/001c.144530 (PMC12579898; doi:10.36469/001c.144530)
Supplement: Online Supplementary Material [file jheor_2025_12_2_144530_308423.pdf]

Journal of Health Economics and Outcomes Research

Online Supplementary Material

Investigation of Differences Between Manufacturers and Public Analyses in Health Technology Assessment in Japan. *JHEOR*. 2025;12(2):173-182. doi:10.36469/jheor.2025.144530

Table S1: List of Target Products

Table S2: Breakdown of Consistency and Inconsistency in Assessments of Additional Benefits As Well As Outcome Measures and Analysis Methods Used To Support Assessments Over Time

Table S3: Inclusion of ITCs for Orphan and Non-orphan Drugs in Manufacturers' and C2H Analyses (Excluding Products With Comparative Trials Against HTA-Designated Comparators for Regulatory Approval)

This supplementary material has been provided by the authors to give readers additional information about their work.

This is an open-access article distributed under the terms of the Creative Commons Attribution 4.0 International License (CCBY-4.0). View this license's legal deed at http://creativecommons.org/licenses/by/4.0 and legal code at http://creativecommons.org/licenses/by/4.0/legalcode for more information.

Table S1. List of Target Products

Generic Name	Brand Name	HTA Classification	Indication	Orphan/ Intractable Disease	Date of HTA Designation	Date of Evaluation Decision
Fluticasone furoate/ umeclidinium/ vilanterol	Trelegy 100 Ellipta	H1	Relief of symptoms of chronic obstructive pulmonary disease (chronic bronchitis and emphysema) (when combination therapy with inhaled corticosteroids, long-acting muscarinic antagonists, and long-acting β2 agonists is required)	–	2019/5/15	2021/3/24
Tisagenlecleucel	Kymriah	H3	1. Relapsed or refractory CD19-positive B-cell acute lymphoblastic leukemia<br>2. Relapsed or refractory diffuse large B-cell lymphoma	Orphan	2019/5/15	2021/3/24
Ravulizumab	Ultomiris	H1	Paroxysmal nocturnal hemoglobinuria	Orphan and intractable disease	2019/8/28	2021/4/14
Vortioxetine	Trintellix	H1	Depression and depressive states	–	2019/11/13	2021/5/26
Ivabradine	Coralan	H2	Chronic heart failure with sinus rhythm and resting heart rate ≥75 bpm at treatment initiation (only for patients receiving standard treatment including β-blockers)	–	2019/11/13	2021/5/26
Posaconazole	Noxafil	H1	1. Prevention of deep fungal infections in patients undergoing hematopoietic stem cell transplantation or with expected neutropenia due to hematologic malignancies<br>2. Treatment of the following fungal infections: fusariosis, mucormycosis, coccidioidomycosis, chromoblastomycosis, and mycetoma	–	2020/4/8	2021/11/10
Trastuzumab deruxtecan	Enhertu	H1	1. Unresectable or recurrent HER2-positive breast cancer with prior chemotherapy (only when standard treatment is difficult)<br>2. Unresectable advanced or recurrent HER2-positive gastric cancer that has progressed after chemotherapy	–	2020/5/13	2022/3/23
Cabozantinib	Cabometyx	H1	1. Unresectable or metastatic renal cell carcinoma<br>2. Unresectable hepatocellular carcinoma that has progressed after chemotherapy	–	2020/5/13	2022/5/18
Semaglutide	Rybelsus	H1	Type 2 diabetes mellitus	–	2020/10/11	2022/7/20
Galcanezumab	Emgality	H1	Prevention of migraine attacks	–	2021/4/14	2023/1/18
Polatuzumab vedotin	Polivy	H1	Diffuse large B-cell lymphoma	Orphan	2021/5/12	2022/11/9
Amikacin sulfate	Arikayce	H1	[Indicated bacteria]: *Mycobacterium avium* complex (MAC) susceptible to amikacin<br>[Indication]: Nontuberculous mycobacterial pulmonary disease caused by MAC	–	2021/5/12	2022/12/14

Table S1. List of Target Products

Generic Name	Brand Name	HTA Classification	Indication	Orphan/ Intractable Disease	Date of HTA Designation	Date of Evaluation Decision
Daratumumab/ vorhyaluronidase alfa	Darzquro	H1	1. Multiple myeloma<br>2. Systemic AL amyloidosis	Orphan and intractable disease[a]	2021/5/12	2023/2/15
Teduglutide	Revestive	H2	Short bowel syndrome	Orphan	2021/8/4	2023/1/18
Remdesivir	Veklury	H1	Infection caused by SARS-CoV-2	–	2021/8/4	2023/1/18
Selpercatinib	Retevmo	H1	1. Unresectable advanced or recurrent non-small cell lung cancer with RET fusion gene<br>2. Unresectable thyroid cancer with RET fusion gene	Orphan	2021/11/17	2023/8/23
Enfortumab vedotin	Padcev	H1	Unresectable urothelial carcinoma that has progressed after chemotherapy	–	2021/11/17	2023/2/15
Gefapixant citrate	Lyfnua	H1	Refractory chronic cough	–	2022/4/13	2023/6/14
Bimekizumab	Bimzelx	H1	Diseases insufficiently controlled by existing treatments: plaque psoriasis, pustular psoriasis, and erythrodermic psoriasis	–	2022/4/13	2023/9/13
Clazosentan sodium	Pivlaz	H1	Cerebral vasospasm after subarachnoid hemorrhage due to cerebral aneurysm, and associated cerebral infarction and ischemic symptoms	–	2022/4/13	2023/9/13
Efgartigimod alfa	Vyvgart	H1	Generalized myasthenia gravis (only when corticosteroids or other immunosuppressants are insufficiently effective)	Orphan and intractable disease	2022/4/13	2023/10/11
Andexanet alfa	Ondexxya	H2	Reversal of anticoagulant effects in life-threatening or uncontrolled bleeding in patients receiving direct oral Factor Xa inhibitors (apixaban, rivaroxaban, or edoxaban)	Orphan	2022/5/18	2023/10/11
Valbenazine tosilate	Dysval	H2	Tardive dyskinesia	–	2022/5/18	2023/10/11
Finerenone	Kerendia	H1	Chronic kidney disease with type 2 diabetes mellitus (excluding patients with end-stage renal disease or on dialysis)	–	2022/5/18	2024/3/13
Molnupiravir	Lagevrio	H1	Infection caused by SARS-CoV-2	–	2022/8/10	2024/3/13
Deucravacitinib	Sotyktu	H1	Diseases insufficiently controlled by existing treatments: plaque psoriasis, pustular psoriasis, and erythrodermic psoriasis	–	2022/11/9	2024/4/24
Tezepelumab	Tezspire	H1	Bronchial asthma (only for severe or refractory cases not controlled by existing treatments)	–	2022/11/9	2024/6/12
Tirzepatide	Mounjaro	H1	Type 2 diabetes mellitus	–	2023/3/8	2024/9/11
Ensitrelvir fumaric acid	Xocova	H1	Infection caused by SARS-CoV-2	–	2023/3/8	2024/10/9
Ropeginterferon alfa-2b	Besremi	H1	Polycythemia vera (only when existing treatments are insufficient or inappropriate)	–	2023/5/17	2024/10/9
Ritlecitinib tosilate	Litfulo	H1	Alopecia areata (only in refractory cases involving extensive areas of hair loss)	–	2023/8/23	2025/1/29

Abbreviation: HTA, health technology assessment.
[a]Only for systemic AL amyloidosis.

Table S2. Breakdown of Consistency and Inconsistency in Assessments of Additional Benefits As Well As Outcome Measures and Analysis Methods Used To Support Assessments Over Time

	N	Consistent Outcome Measures and Analysis Methods	Inconsistent Outcome Measures and Analysis Methods
Entire period (31 products)			
Analysis populations (N = 72)[a]			
Analysis population for which the assessments of additional benefits are consistent between the manufacturers and C2H	56	66.1%	33.9%
Analysis population for which the assessments of additional benefits are inconsistent between the manufacturers and C2H	16	6.3%	93.7%
Early period (17 products designated April 2019–March 2022)			
Analysis populations (N = 49)[a]			
Analysis population for which the assessments of additional benefits are consistent between the manufacturers and C2H	39	74.4%	25.6%
Analysis population for which the assessments of additional benefits are inconsistent between the manufacturers and C2H	10	10.0%	90.0%
Late period (14 products designated April 2022–March 2025)			
Analysis populations (N = 23)			
Analysis population for which the assessments of additional benefits are consistent between the manufacturers and C2H	17	47.1%	52.9%
Analysis population for which the assessments of additional benefits are inconsistent between the manufacturers and C2H	6	0%	100%

Abbreviation: C2H, Center for Outcomes Research and Economic Evaluation for Health.
[a]Two analysis populations deemed unanalyzable by both the manufacturers and C2H were excluded.

Table S3. Inclusion of ITCs for Orphan and Non-orphan Drugs in Manufacturers' and C2H Analyses (Excluding Products With Comparative Trials Against HTA-Designated Comparators for Regulatory Approval)

Inclusion of ITC (%)	Manufacturers	C2H[a]	C2H vs Manufacturers[b]
Orphan drugs (N = 4)	100% (4/4 products)	50.0% (2/4 products)	50.0% (2/4 products)
Orphan drugs excluding those for designated intractable diseases (N = 3)	100% (3/3 products)	66.7% (2/3 products)	66.7% (2/3 products)
Non-orphan drugs (N = 13)	61.5% (8/13 products)	61.5% (8/13 products)	87.5% (7/8 products) [c]

Abbreviations: C2H, Center for Outcomes Research and Economic Evaluation for Health; HTA, health technology assessment; ITC, indirect treatment comparison.
[a]Products were included where C2H either accepted the methods and results of ITCs conducted by the manufacturers or conducted ITCs independently.
[b]Proportion of products for which C2H either accepted the manufacturers' ITCs or conducted them independently, among those where the manufacturers included such analyses.
[c]The denominator represents the number of products for which ITCs were conducted in the manufacturers' analyses. One product was not included in the numerator, for which C2H conducted an ITC while the manufacturer did not.
